# Deep neural network enabled active metasurface embedded design

**DOI:** 10.1515/nanoph-2022-0152

**Published:** 2022-06-10

**Authors:** Sensong An, Bowen Zheng, Matthew Julian, Calum Williams, Hong Tang, Tian Gu, Hualiang Zhang, Hyun Jung Kim, Juejun Hu

**Affiliations:** Department of Materials Science & Engineering, Massachusetts Institute of Technology, Cambridge 02139, MA, USA; Department of Electrical & Computer Engineering, University of Massachusetts Lowell, Lowell 01854, MA, USA; Booz Allen Hamilton Inc, Arlington 22203, VA, USA; Department of Physics, University of Cambridge, Cambridge CB3 0HE, UK; Materials Research Laboratory, Massachusetts Institute of Technology, Cambridge 02139, MA, USA; NASA Langley Research Center, Hampton 23681-2199, VA, USA

**Keywords:** active metasurface, deep neural network, embedded design, phase change material, tunable metasurface

## Abstract

In this paper, we propose a deep learning approach for forward modeling and inverse design of photonic devices containing embedded active metasurface structures. In particular, we demonstrate that combining neural network design of metasurfaces with scattering matrix-based optimization significantly simplifies the computational overhead while facilitating accurate objective-driven design. As an example, we apply our approach to the design of a continuously tunable bandpass filter in the mid-wave infrared, featuring narrow passband (∼10 nm), high quality factors (*Q*-factors ∼ 10^2^), and large out-of-band rejection (optical density ≥ 3). The design consists of an optical phase-change material Ge_2_Sb_2_Se_4_Te (GSST) metasurface atop a silicon heater sandwiched between two distributed Bragg reflectors (DBRs). The proposed design approach can be generalized to the modeling and inverse design of arbitrary response photonic devices incorporating active metasurfaces.

## 1 Introduction

Metasurfaces are the 2-D version of metamaterials and serve as a versatile platform for building ultra-thin and large-scale devices. By carefully engineering the geometry and material of meta-atoms, the building blocks of metasurfaces, we can realize independent phase and amplitude control of the optical wavefront at the subwavelength level [1–11]. Conventional metasurface designs constructed with passive metallic [1, 12, 13] or all-dielectric [2, 3] materials require re-design of meta-atoms to change their optical functions. In contrast, active metasurfaces building on tunable materials enable dynamic reconfiguration to adapt to different tasks. Phase change materials (PCMs) such as Ge_2_Sb_2_Te_5_ (GST) [14–22] or Ge_2_Sb_2_Se_4_Te (GSST) [23–28] in particular present an attractive all solid-state material platform for active metasurfaces benefiting from their remarkably large index modulation upon amorphous–crystalline structural transformation. The material refractive index change allows different resonant modes to be excited in the meta-atom structure, thereby realizing tuning of its electromagnetic responses.

Despite the exciting prospects of active metasurfaces, they are also confronted with several important challenges. It is nontrivial to obtain high-*Q* resonances with metasurfaces, which mandates judicious engineering of coupling to free-space radiative channels while minimizing internal material losses [29]. This requirement becomes far more challenging when active tuning over a large spectral range is necessary, since the high-*Q* condition is often wavelength sensitive. Similar constraints also apply to tuning metasurface responses in the angular domain. Further, sufficient out-of-band rejection and mitigation of undesired transmission/reflection sidebands outside the operating wavelength range prove challenging.

These barriers can be overcome by combining metasurfaces with other classes of photonic structures to impart the desired characteristics. One case in point is an optical bandpass filter comprising a metasurface embedded in a Fabry–Perot (F–P) cavity [30]. This design has several advantages over single-layer metasurfaces: the coupling to radiative modes can be modulated by changing the distributed Bragg reflector (DBR) pair number; the structure suppresses out-of-band transmission within the photonic bandgap (stopband) of the DBR which improves filter extinction ratio; and, the sidebands can be eliminated via apodization. Compared to traditional multilayer F–P cavities (interference filters), the introduction of a metasurface enables facile engineering of the dispersion characteristics far beyond what a simple homogenous layer can attain [31]. Design of such active metasurface embedded structures, however, can be a computation-intensive task given the massive number of degrees of freedom encompassing free-form meta-atom geometries and F–P cavity layer structures collectively operating over a continuum of tunable material states.

In recent years, deep learning approaches and deep neural networks (DNNs) have been investigated as a solution to handle such complex photonic design problems [32–58], including the design of multilayer structures [39, 42, 54, 55], freeform meta-atoms [32, 56, 59] and diffractive imagers [60]. In these examples, DNNs were constructed to directly predict the transmission or reflection spectra of passive photonic structures. In this paper, we expand the repertoire of deep-learning-based photonic design by showing that DNNs can also be implemented to both predict and inverse design broadband complex *S*-parameters of active metasurface elements. The DNNs can then be used in conjunction with transfer matrix method (TMM) based analytical optimization to facilitate computationally efficient design of metasurface-embedded tunable photonic devices.

To illustrate the utility of our hybrid design approach, we designed an actively tunable optical bandpass filter integrating a PCM metasurface with two DBRs operating in the mid-wave infrared (MWIR) waveband. Such a filter is currently being explored for space-borne multispectral imaging and sensing applications [61, 62] given their substantial size, weight, and power (SWaP) advantages in comparison to conventional motorized filter wheels. The PCM metasurface is constructed with a periodic array of GSST meta-atoms atop a doped Si-on-SiO_2_ substrate, and the DBRs are composed of quarter-wavelength-thick a-Si and SiO_2_ layers. By applying voltage pulses to the doped silicon heater underneath the meta-atoms to electrothermally trigger structural transition of GSST, the optical phase delay between the two DBRs and hence the center wavelength of the transmission band can be actively tuned [23, 63, 64]. Compared to a traditional F–P structure with a planar PCM cavity layer [61], this design not only enables versatile dispersion engineering but also offers a practical architecture for electrical reversible switching of PCMs. By singulating a PCM film into discrete meta-atoms surrounded by a thermally conductive capping layer (MgF_2_ in this case), we can significantly expedite heat extraction during the PCM amorphization process to achieve uniform switching throughout the entire PCM volume [65]. The proposed fabrication process of the device is described in the Supplementary Information Section I and experimental realization is the subject for future studies.

## 2 Design method

**Figure 1:**
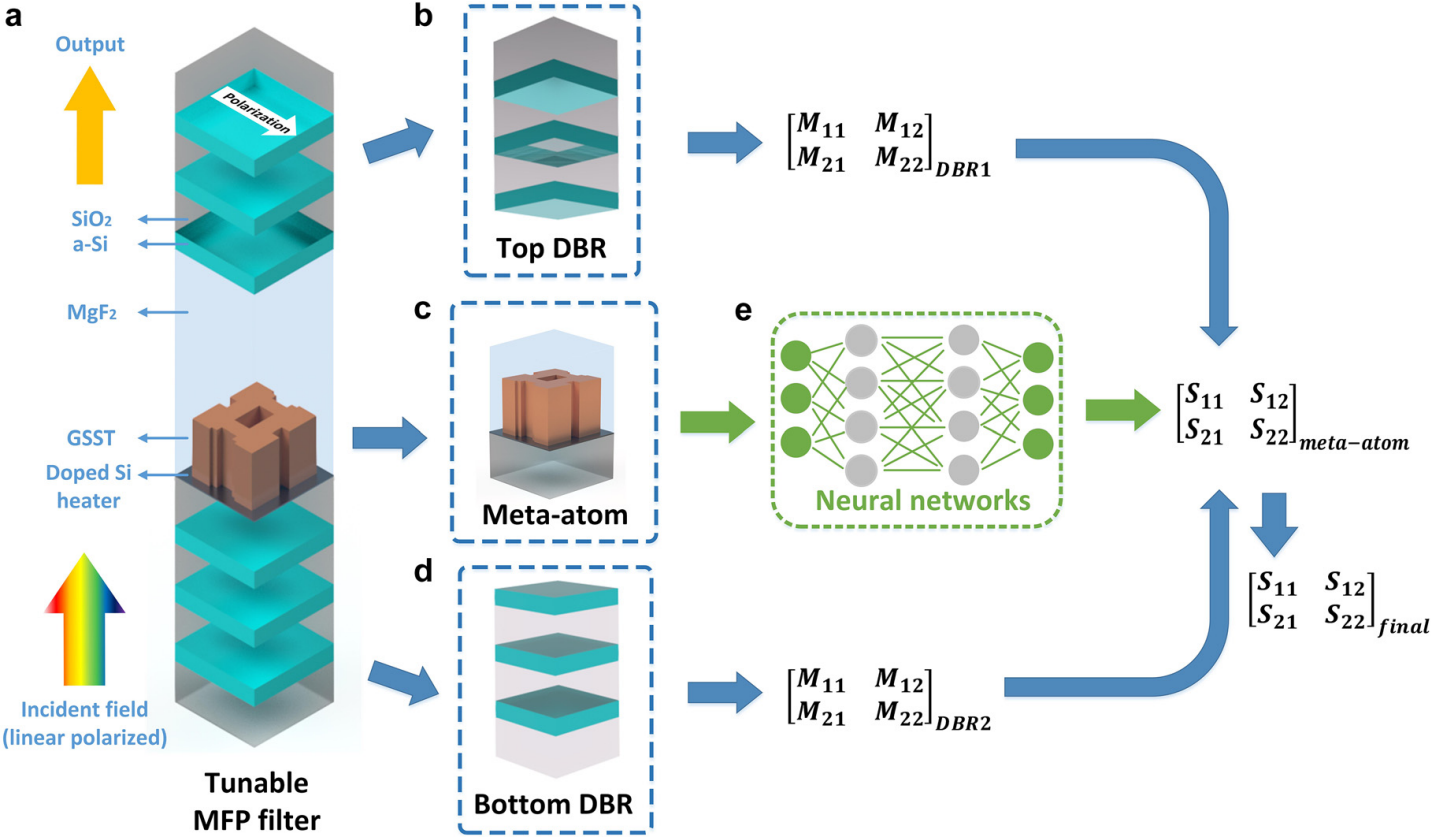
MFP filter design methodology. (a) A schematic diagram of the tunable filter structure. (b) The top DBR. (c) The GSST meta-atom in the center of the cavity. (d) The bottom DBR. (e) The forward prediction DNN to evaluate the *S*-matrix of the meta-atom. The *S*-matrices were then translated into transfer matrices and multiplied with the transfer matrices of the DBRs to calculate the final *S*-matrix of the whole structure.

The metasurface-embedded F–P filters (hereinafter referred to as the “MFP filters”) were constructed with two DBRs and one metasurface layer sandwiched in-between, i.e., acting as a controllable cavity (Figure 1a). Each meta-atom element (Figure 1c) consists of a freeform GSST structure sitting on a square-shaped SiO_2_ substrate. Between the substrate and the GSST material, one thin layer (30 nm) of doped silicon was also added [66] which acts as an electrically controlled resistive heater. By carefully engineering the amplitude and duration of the voltage pulses applied to the heater, the GSST material can be dynamically switched between amorphous, partially crystalline, or fully crystalline states [63, 64]. Both the top and the bottom DBR consists of pairs of a-Si–SiO_2_ (high-low index) films, and the pair numbers are analytically optimized using TMM. The meta-atoms are encapsulated in MgF_2_, whose thickness can be tuned to adjust the cavity length. Without loss of generality, the spectra of interest were set to be from 60 to 100 THz (3–5 µm in wavelength), with a center wavelength of 4 µm. The incident light is a linearly-polarized plane wave illuminating from the substrate side. Considering the electronically large structure size (usually several wavelengths in the propagating direction) and the resonant nature of this MFP filters, full-wave simulation of the entire MFP filter structure is usually time-consuming. Therefore, exhausting the full design space—including tunable refractive indices of the GSST material, thickness of the DBR layers, spacing between DBRs, lattice size, as well as the geometry of freeform meta-atoms—by parametric sweep alone to find the globally optimal design is computationally unfeasible. Instead, we resorted to the TMM approach. As shown in Figure 1, the transfer matrices of the top DBR (Figure 1b), center meta-atom (Figure 1c), and bottom DBR (Figure 1d) can be separately calculated and multiplied together subsequently to obtain the total transfer matrix. The spectral response of the entire transmissive MFP filter can be derived by converting its total transfer matrix into an *S*-matrix (more details are included in Supplementary Information Section II). Due to the near-field effects and coupling between the freeform meta-atom and its surroundings, the *S*-matrix of the meta-atom cannot be numerically analyzed, which limits the modeling efficiency of this TMM approach. To tackle this challenge, we trained a forward prediction DNN (Figure 1e) that generates accurate *S*-parameters of GSST meta-atoms given its dimensions and crystallization state. Since the DNNs calculate the output on a one-time-calculation basis, optical performance of the MFP filters can be predicted with minimal time cost, which further accelerates their optimization and inverse design.

## 3 Forward prediction DNN

**Figure 2:**
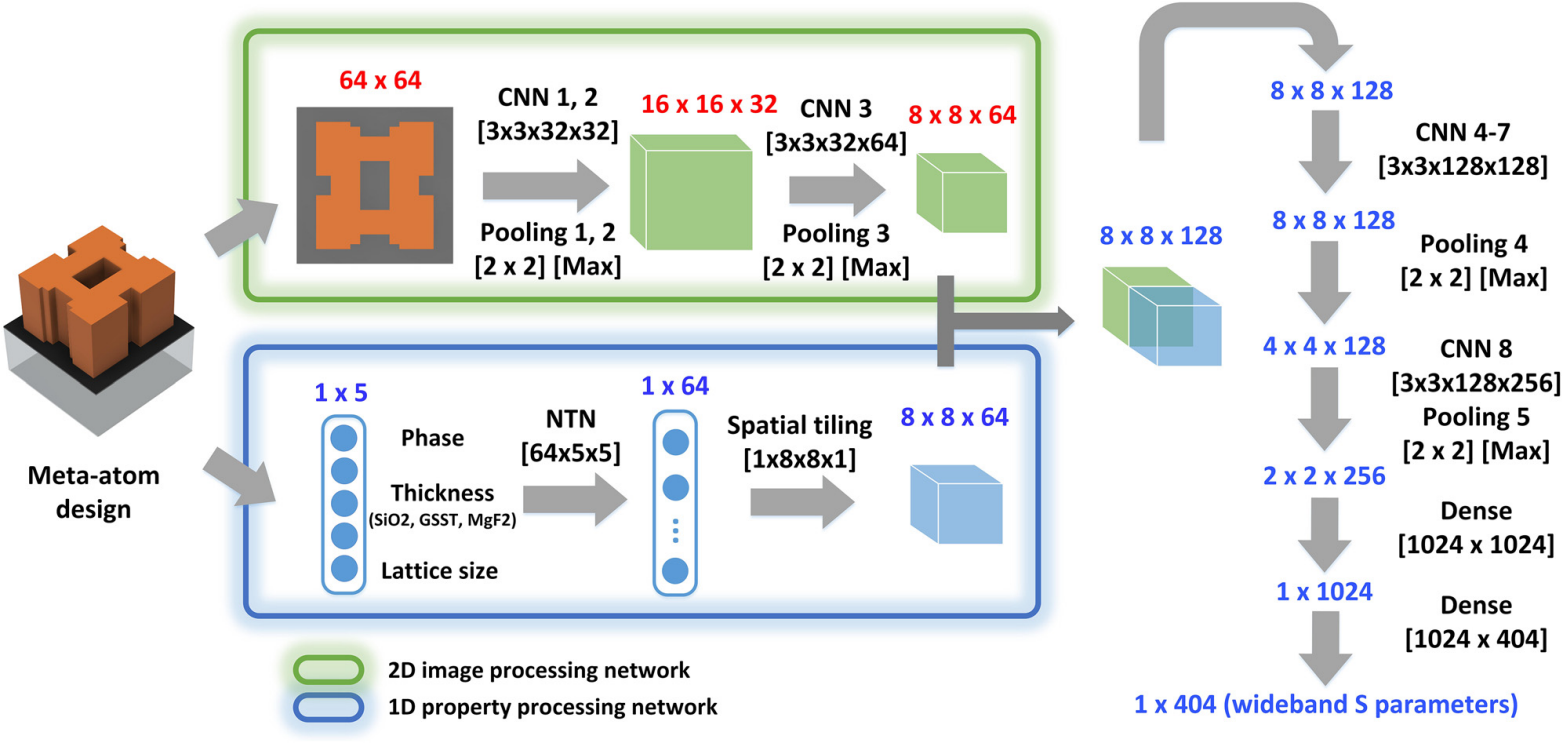
Network architecture. The meta-atom design parameters were evaluated using a 2-D image processing network (circled in green) and a 1-D property processing network (circled in blue). After processing with several convolutional layers and dense layers, the *S*-matrix (real and imaginary part) of the meta-atom over the spectrum of 60–100 THz were generated as the final output. 101 frequency points, with a spacing of 0.04 THz, were used to represent the full spectra for each *S*-parameter.

A forward prediction DNN was constructed and trained to achieve fast and accurate evaluation of meta-atoms shown in Figure 1c. The forward prediction DNN was constructed based on a convolutional neural network (CNN) [67] architecture (Figure 2). The meta-atom designs can be described with two sets of parameters: the 2-D cross-section of the meta-atom and other properties, including: the crystallization fraction of GSST (between “0” representing amorphous and “1” corresponding to fully crystalline); thicknesses of the SiO_2_ layer between doped silicon heater and the bottom DBR; the height of the GSST meta-atom and the thickness of the MgF_2_ capping layer; and the lattice size (period) of the meta-atoms. The 2-D cross-sections were processed through a 2-D image processing network (circled in green in Figure 2), which is composed of three consecutive convolutional layers. The rest of the meta-atom’s properties were processed with a 1-D property processing network (circled in blue in Figure 2), which includes a Neural Tensor Network (NTN) [32, 68]. The NTN relates the input parameters multiplicatively instead of only implicitly, which effectively accelerates the training process when the relationship between input and output is highly nonlinear. Specifically, the output of this NTN layer is given by:

(1) $$Output=f\left(e^{T}W^{\left[1:k\right]}e+Ve+b\right)$$

where *e* represents the 1-D property of the meta-atom and *W*, *V* and *b* represents the weight and bias, respectively. The output of the NTN was then spatially tiled and concatenated with the output of the 2-D image processing network. The combined output was then further processed with more convolutional layers, during which more high dimensional hidden features of the original meta-atom were revealed and extracted. Finally, two dense layers translated the extracted features into the wideband *S*-parameters of the meta-atom. Since the meta-atom can be treated as a two-port network, the final output is composed of wideband *S*_11_, *S*_12_, *S*_21_, and *S*_22_ responses. Two prediction networks with the same architecture (as shown in Figure 2) were trained to predict the real and imaginary parts of the complex *S*-parameters of the meta-atoms, respectively. Detailed hyperparameters are included in the Supplementary Information Section III.

**Figure 3:**
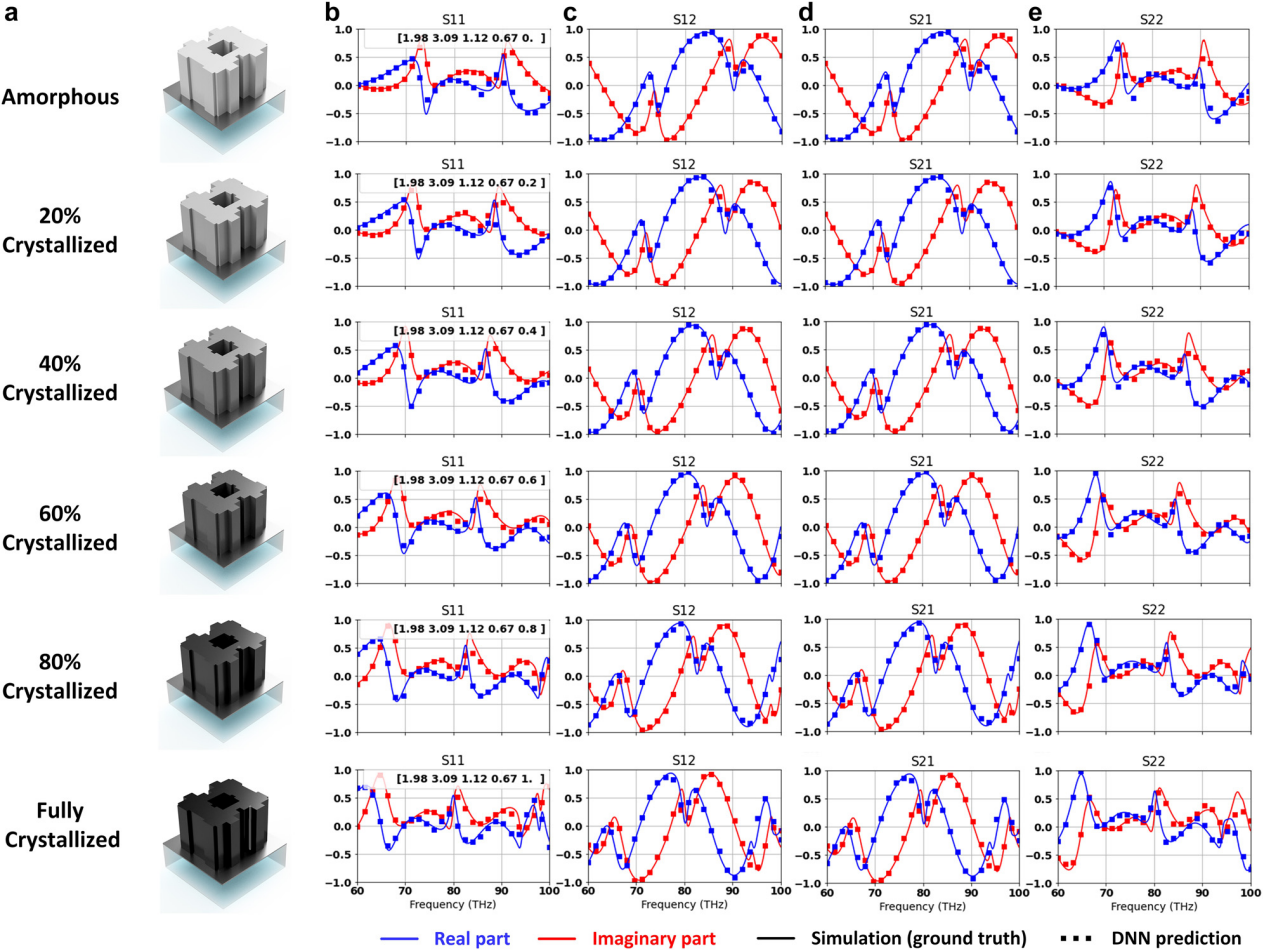
*S*-matrix prediction using forward DNN. (a) Meta-atoms in different crystallization states. 3-D model of each meta-atom is shown on the left. (b) *S*_11_ plots. (c) *S*_12_ plots. (d) *S*_21_ plots. (e) *S*_22_ plots. Dotted lines represent the DNN prediction results, while solid curves are simulation results (ground truth). Parameters including lattice size, the thicknesses of MgF_2_, GSST meta-atom, and SiO_2_ substrate, as well as the crystallization states are shown on the top-right corner of the *S*_11_ subplots (parameters listed in the same order as here; all lengths in μm). Additional examples of DNN prediction accuracy are included in Supplementary Information Section IV.

To train the network, over 10^5^ groups of meta-atoms with quasi-freeform 2D cross-sections were randomly generated using the “needle drop” approach [69]. Several rectangular bars, with a minimum generative resolution of 1 pixel, were randomly generated and placed together within a square canvas (64 × 64 pixels) to form random patterns. To accelerate the pattern generation and data collection process, the patterns in the top left quadrant of each unit cell were generated first and then symmetrically replicated along both *x* and *y* axis to form the complete patterns. The other parameters including the state of the PCM and the dimensions of the meta-atoms were created randomly within the following range (with lengths all in µm): material state $\in \left[0,1\right]$ , SiO_2_ substrate thickness $\in \left[0.6,0.8\right]$ , GSST thickness $\in \left[0.5,2\right]$ , MgF_2_ thickness $\in \left[1,3\right]$ , lattice size $\in \left[0.5,1.5\right]$ , since these ranges provides ample samples of the meta-atoms’ *S*-parameters. The *S*-matrices of these randomly generated designs were evaluated using the full-wave simulation tool CST Studio Suite. The 2-D cross sections of the meta-atoms, along with the 1-D properties were assigned as the input of the network, while their corresponding wideband *S*-matrices were designated as the outputs. Among the 10^5^ groups of training data, 70% were used during the training process, the remaining 30% were used to evaluate the fully-trained network. The parameters of the hidden layers shown in Figure 2 were optimized during the training to minimize the difference between predicted results and the ground truth. The training was performed on a workstation consists of a 16-core CPU with 4.7 GHz clock speed and a NVidia 1080Ti GPU. Both DNNs converged after 72 h of training. Upon completion of the training, the average mean square error (MSE) was 7.3 × 10^−4^ for the real part and 7.8 × 10^−4^ for the imaginary part of the complex *S*-parameters. To showcase the DNN’s accuracy, we randomly selected a meta-atom design from the test dataset and employed the prediction network to evaluate its performance in different crystallization states (Figure 3). The real part (in blue) and imaginary parts (in red) of their complex *S*-matrices, including the *S*_11_, *S*_12_, *S*_21_, and *S*_22_ are shown on the right in each subplot. The dotted lines are the prediction results generated by the network, while the solid curves are the ground truth derived with the full-wave simulation tool. Among the 101 sample points that were used across 60–100 THz, only 26 were plotted in each subplot in Figure 3 for the sake of clarity. Due to optical reciprocity, *S*_12_ and *S*_21_ for all meta-atoms are identical. In practice, we found out that removing one of these two *S*-parameters from the output did not further increase the training accuracy, and thus all *S*-parameters are simultaneously predicted for simplicity. As indicated by the small training error, the prediction results agreed well with the ground truth in all cases.

Using the TMM approach in Figure 1, the spectral responses of an MFP filter structure can be efficiently calculated (in milliseconds with a single CPU workstation). This forward DNN has two major advantages: (1) due to its high computational efficiency, this forward DNN can be adopted to evaluate the performance of the designs in closed-loop optimization algorithms, which otherwise would have been the most time-consuming process (e.g., the full-wave simulation of one meta-atom in Figure 3 could take minutes with a single CPU workstation) during the optimization, and (2) since we are building this forward prediction DNN in a way that it can handle meta-atoms with different design parameters, the network can be employed to quickly assemble sub-datasets with less degrees of freedom for more specific design problems. For example, since most metasurface and meta-devices are composed of elements with the same lattice size and thickness, we can use the prediction network to choose the right parameter combinations (including the lattice size, thickness, and material indices) that leads to the largest modulation depth of phase and amplitude [69]. Besides, given the fabrication complexity associated with freeform meta-atoms which entail small critical dimensions [40, 52, 56], we can utilize the forward DNN to predict the spectral responses of a class of meta-atoms with simple shapes such as rectangles [70] and “H’s”. (3) These patterns can be easily described using a limited number of parameters, which largely reduces the difficulty of inverse design [32, 33, 38, 39, 42, 43, 46, 53, 58]. In the following section, we demonstrate how to construct and train an MFP filter inverse design network with a sub-dataset generated by the forward prediction DNN which is composed of only “H”-shaped meta-atoms.

## 4 Inverse design DNN

**Figure 4:**
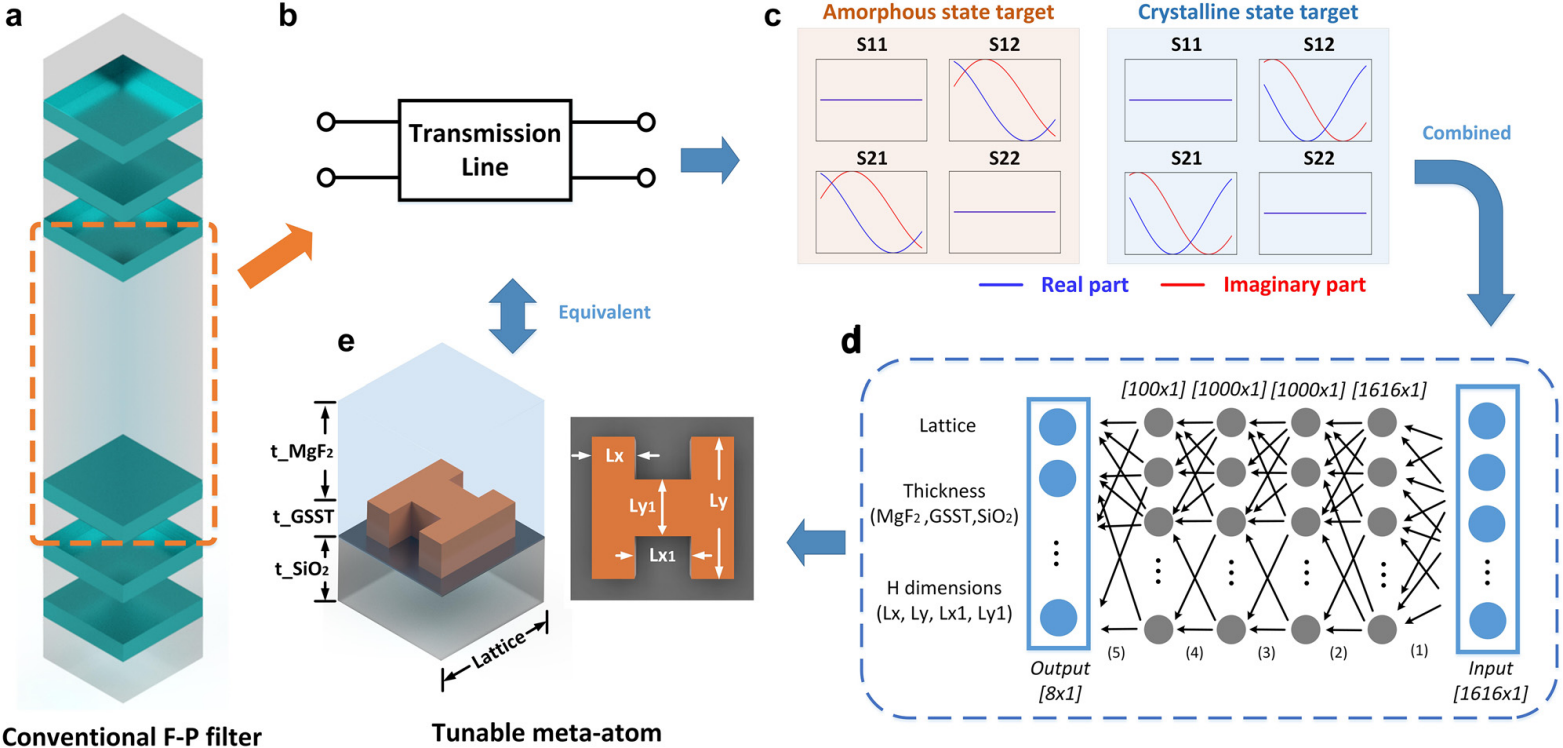
Inverse design DNN. (a) A conventional F–P bandpass filter with two DBRs separated by a cavity. (b) The spacing (cavity) between two DBRs was treated as a two-port transmission line. (c) *S*-matrices of the transmission lines were designated as input of the inverse DNN. (d) The meta-atom inverse DNN constructed based on fully-connected neural networks. (e) Output of the inverse DNN, which is a combination of design parameters including all meta-atom dimensions.

With meta-atoms constructed with PCMs such as GSST, if we found a specific design with high transmission and large phase delay tuning range when it is in different crystallization states, then it is possible to realize tuning of F–P resonances inside the MFP filter structure at will. Specifically, for bandpass filters with a given tuning range, we can calculate the target electrical length of the spacing between two DBRs at two ends of the tuning range (Figure 4a), and then realize a similar optical response of the shorter electrical length using one GSST meta-atom design in the amorphous state, and likewise use the same design in the fully crystalline state to attain the longer electrical length (Figure 4b). The continuous tuning of the F–P resonances between these two states can be achieved by manipulating the crystallization state of the GSST material through adjusting voltage pulse parameters [63, 64]. Inspired by this idea, we constructed an inverse design DNN for the design of actively tunable filters with given tuning range targets and filter functions (edge, bandpass, etc.). As a proof of concept, we focused on the bandpass filters across the MWIR band. As shown in Figure 4c, the target wideband *S*-matrices (in both the amorphous and the fully crystalline states) were assigned as the inputs of the inverse DNN while the shape and dimensions of the corresponding meta-atoms were defined as the output. Since the meta-atoms with the shape of letter “H” provides sufficient transmission and phase delay responses while maintaining a low fabrication difficulty comparing to the freeform shapes [3, 69], we used the “H-shaped” meta-atoms to assemble the training dataset for the inverse DNN. Specifically, we constructed “H-shaped” patterns with random dimensions on a 64 × 64 canvas, then assigned the 2-D “H-shaped” patterns with four parameters (*Lx*, *Ly*, *Lx*_1_, *Ly*_1_, as shown in Figure 4d) and combined these four parameters with the other dimensions (*t*_MgF2_, *t*_GSST_, *t*_SiO2_,* lattice*) that were randomly created within the preset data range mentioned above. The *S*-matrices of these randomly-generated meta-atoms were evaluated with the fully-trained prediction DNN. The final training dataset contains over 5 × 10^4^ groups of “H-shaped” meta-atom designs, along with their wideband *S*-matrices within the 60–100 THz range in both amorphous and crystalline states.

**Figure 5:**
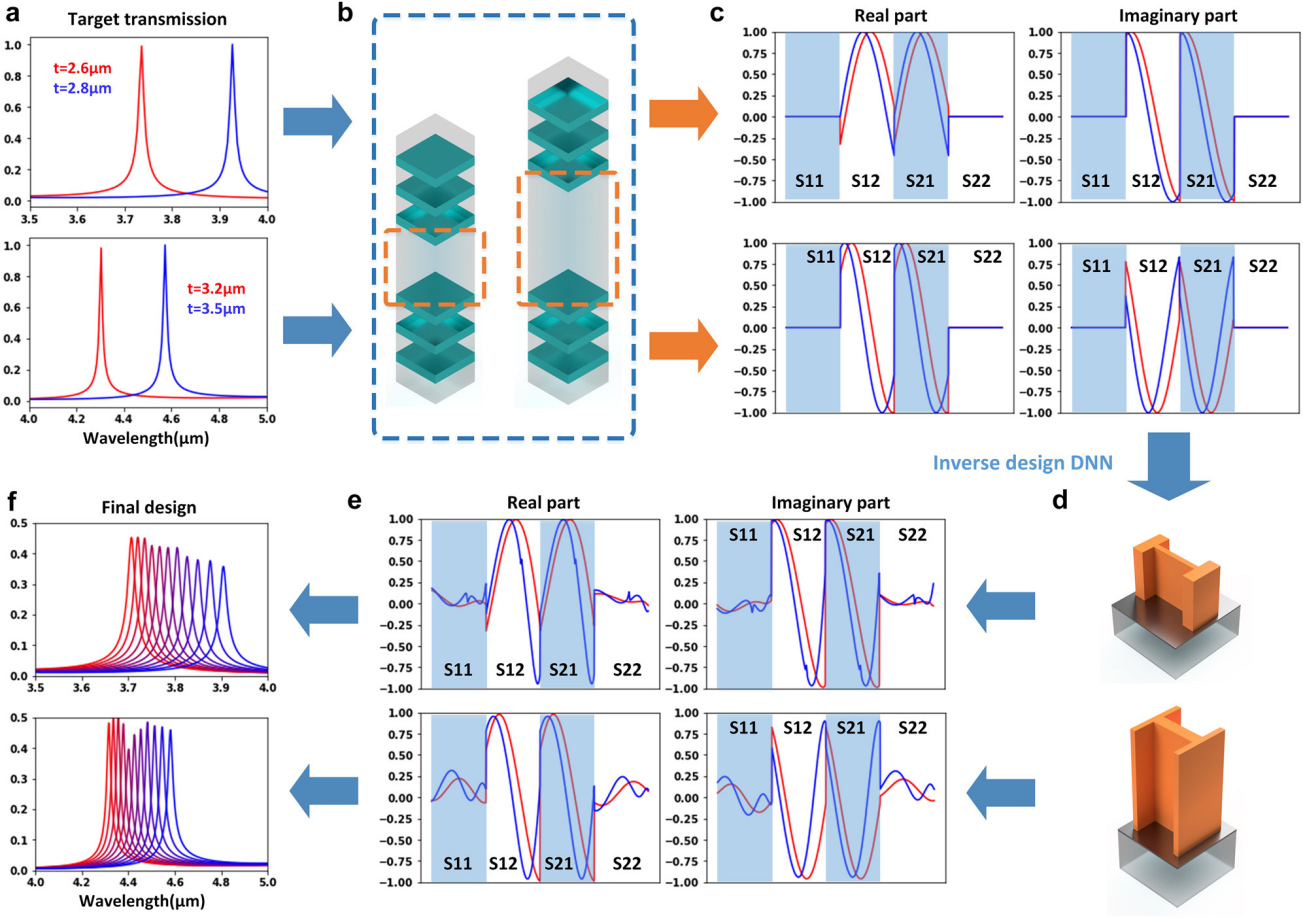
Design examples with the inverse DNN. (a) Target transmission spectra. The positions of the two peaks (red represents target response in the amorphous state, blue represents target response in the crystalline state), along with the tuning range were defined. (b) The distance between DBRs (in both states) was calculated based on the target spectrum. (c) Corresponding *S*-matrices (amplitude vs. frequency, 60–100 THz) were calculated and fed into the inverse DNN. (d) Design parameters generated by the inverse DNN. (e) *S*-matrices (amplitude vs. frequency, 60–100 THz) of the generated designs showing in (d). (f) Transmission spectra of the final design. Red and blue curves represent the transmission of the filter design in the amorphous and the fully crystalline state. The other 9 curves represent the responses of the design in intermediate states between amorphous and fully crystalline.

After the inverse DNN in Figure 4c was fully trained, we employed this network to design two transmissive tunable bandpass filters with different tuning ranges. The top and bottom DBRs each consist of 3 pairs of a-Si (*n* = 3.4) and SiO_2_ (*n* = 1.39) films. Taking *λ* = 4 µm as the center wavelength of the bandpass, the thicknesses of the quarter-wavelength a-Si and SiO_2_ layers were set to 294 and 719 nm, respectively. As shown in Figure 5a, the first design target has a tuning range from 3.75 to 3.95 µm, which requires the distance between DBRs (Figure 5b) changing from 2.6 to 2.8 µm. *S*-matrices of this meta-atom between the 60–100 THz frequency range, including *S*-parameters in both amorphous state (in red) and fully crystalline state (in blue) were fed to the inverse DNN as inputs. The *S*-matrices of the generated design (Figure 5d) are very similar to the preset targets (Figure 5e). The transmission spectra of the final MFP filter design (Figure 5f) are therefore close to the design objective (Figure 5a). For the second design (showing at the bottom of each subplot), the center wavelength tuning range was set to be $\in \left[4.3\;\mu m,4.6\;\mu m\right]$ . Similarly, the optical performance of the design generated by the inverse DNN are similar to the design target. It is worth noticing that due to the nonzero *S*_11_ and *S*_22_ values of the meta-atom designs, this multilayer filter design was not perfectly matched at the interfaces between the meta-atom and the DBRs, which leads to a certain amount of reflected energy at resonances. This mismatch, along with the unneglectable loss of the GSST material and the doped silicon heaters, has limited peak transmission (<50% and decreased as the material switched to fully crystalline) of the designs (Figure 5f). It is worth mentioning that the highly resonant responses showing in Figure 5f have further justified the necessity of our TMM-DNN approach, since the large prediction errors caused by abrupt changes in the spectral responses (peaks, dips and phase wrappings) tend to be averaged among large number of sample points that used to sketch the whole spectra, meaning it is difficult for the DNNs to predict the accurate resonant-type responses (as have been reported in previous literature [32]). Comparing to the metasurface embedded devices, there are less resonances in the metasurfaces, making the wideband *S*-parameters of metasurfaces easier to predict with the TMM-DNN approach. One thing worth mentioning is that this inverse design method is not limited to the design of “H-shaped” embedded metasurfaces. The network showing in Figure 4d can be easily modified to adapt to the design of tunable meta-atoms that can be described with several parameters. Furthermore, a Generative Adversarial Network [40, 56] can be constructed to generate high performance meta-atoms with complicated (e.g., freeform) shapes, to fully unveil the potential of this active metasurface embedded structure.

## 5 Conclusions

We have developed a design approach for complex photonic structures involving embedded phase-change material metasurfaces inside a multilayer cavity and have applied this methodology to the generation of tunable mid-wave infrared bandpass filters. The design approach decouples the metasurface design (accomplished via DNN) and the multilayer optimization (analytically solved by the TMM method guided with intuitive insights from the coupled mode theory), enabling computationally efficient and yet accurate inverse design of such structures. We believe that the hybrid design scheme can be generalized to other photonic structures incorporating metasurfaces where the *S*-matrix description is applicable, for instance waveguide devices, photonic crystals, and stacked multilayer metasurfaces.

## Supplementary Material

Supplementary Material Details
