# Supplementary material for: Deep neural network enabled active metasurface embedded design
Source: Nanophotonics. 2022 Jun 10;11(17):4149–58. doi: 10.1515/nanoph-2022-0152 (PMC11501697; doi:10.1515/nanoph-2022-0152)
Supplement: Supplementary file 1 — Supplementary Material Details [file j_nanoph-2022-0152_suppl.docx]

**Supporting Information**

Deep neural network enabled active metasurface embedded design

Sensong An^1^, Bowen Zheng^2^, Matthew Julian^3^, Calum Williams^4^, Hong Tang^2^, Tian Gu^1,5^, Hualiang Zhang^2^, Hyun Jung Kim^6^, Juejun Hu^1^

^1^Department of Materials Science & Engineering, Massachusetts Institute of Technology, Cambridge, Massachusetts 02139, USA

^2^Department of Electrical & Computer Engineering, University of Massachusetts Lowell, Lowell, Massachusetts 01854, USA

^3^Booz Allen Hamilton, Arlington, VA 22124 USA

^4^Department of Physics, University of Cambridge, Cambridge, CB3 0HE, UK

^5^Materials Research Laboratory, Massachusetts Institute of Technology, Cambridge, Massachusetts 02139, USA

^6^NASA Langley Research Center, Hampton, VA 23681-2199, USA

In this Supporting Information, we provide further examples to showcase the potential of the proposed inverse design approach. These include phase shifters with different center frequencies and devices with reflection spectrum targets.

1. Fabrication process of the proposed device
2. Details of the transfer matrix-DNN adjoint simulation approach
3. Hyperparameters used in the DNN training process
4. Additional DNN prediction examples
5. Inverse design performance characterization

**Section I – Fabrication process flow of the proposed device**

Doped Si heaters are first fabricated on a silicon-on-insulator (SOI) platform following our previously established protocols (1). Phase-change material (PCM) metasurfaces are first deposited via thermal evaporation and patterned on the chips using electron beam lithography, followed by encapsulation by a sputtered MgF_2_ layer. The backside of the SOI chips is then subjected to an anisotropic KOH wet etch, which self-terminates at the buried oxide layer. The etch step leaves a suspended Si-SiO_2_ membrane structure. Finally, the top and bottom distributed Bragg reflector (DBR) stacks are deposited from the chip front and back sides via magnetron sputtering to complete the fabrication process.

**Section II – Details of the transfer matrix-DNN adjoint simulation approach**

To calculate the spectral responses of the DBR-metasurface-DBR structure, we first calculated the S-matrices of the center meta-atom using the forward prediction DNN:

$$S_{meta-atom}=\left[ \begin{matrix} S_{11} & S_{12} \\ S_{21} & S_{22} \end{matrix} \right]$$

then translated it into the transfer matrix using the following equation:

$$M_{meta-atom}= \left[ \begin{matrix} \frac{\left( 1+S_{11} \right)\left( 1-S_{22} \right)+S_{12}S_{21}}{2S_{21}} & Z_{0}\frac{\left( 1+S_{11} \right)\left( 1+S_{22} \right)-S_{12}S_{21}}{2S_{21}} \\ \frac{1}{Z_{0}}\frac{\left( 1-S_{11} \right)\left( 1-S_{22} \right)-S_{12}S_{21}}{2S_{21}} & \frac{\left( 1+S_{11} \right)\left( 1-S_{22} \right)+S_{12}S_{21}}{2S_{21}} \end{matrix} \right]$$

in which Z_0_ represents the electrical impedance. The transfer matrices of the DBRs can be calculated with:

$$T_{DBR}=\left[ \begin{matrix} cosk_{1}l_{1} & jZ_{1}sink_{1}l_{1} \\ j\frac{1}{Z_{1}}sink_{1}l_{1} & cosk_{1}l_{1} \end{matrix} \right]\times\left[ \begin{matrix} cosk_{2}l_{2} & jZ_{2}sink_{2}l_{2} \\ j\frac{1}{Z_{2}}sink_{2}l_{2} & cosk_{2}l_{2} \end{matrix} \right]\times\ldots$$

where $k=\frac{2\pi}{\lambda}$ represents the wave number in each layer, $Z$ represents the impedance of each layer, and $l$ represents the layer thickness. After deriving the transfer matrices of the two DBR layers, the overall transmission matrices of the device can be calculated by:

$$M_{all}=M_{DBR1}\times M_{meta-atom}\times M_{DBR2}=\left[ \begin{matrix} M_{11} & M_{12} \\ M_{21} & M_{22} \end{matrix} \right]$$

It was subsequently converted back to the S-matrix formalism and we can get:

$$\left[ \begin{matrix} S_{11} & S_{12} \\ S_{21} & S_{22} \end{matrix} \right]_{final}=\left[ \begin{matrix} \frac{M_{11}+M_{12}/Z_{0}-M_{21}Z_{0}-M_{22}}{M_{11}+M_{12}/Z_{0}+M_{21}Z_{0}+M_{22}} & \frac{2(M_{11}M_{22}-M_{12}M_{21})}{M_{11}+M_{12}/Z_{0}+M_{21}Z_{0}+M_{22}} \\ \frac{2}{M_{11}+M_{12}/Z_{0}+M_{21}Z_{0}+M_{22}} & \frac{-M_{11}+M_{12}/Z_{0}-M_{21}Z_{0}+M_{22}}{M_{11}+M_{12}/Z_{0}+M_{21}Z_{0}+M_{22}} \end{matrix} \right]$$

**And the final transmission of the MFP filter can be derived with:**

$$T_{final}= abs(\frac{2(M_{11}M_{22}-M_{12}M_{21})}{M_{11}+M_{12}/Z_{0}+M_{21}Z_{0}+M_{22}})$$

**Section III – Hyperparameters used in the DNN training process**

Hyperparameters used in the training for both forward prediction DNN and the inverse design DNN are shown in Table S1. The hardware consists of a 16-core CPU with 4.7 GHz clock speed, 64 gigabytes of RAM and a NVidia 1080Ti GPU. As shown in the table, after 10,000 iterations, the average test set error stabilized at 0.00073 and 0.00078 for the real and imaginary part, respectively for the forward prediction networks. With the current hardware setup, the training takes 72 hours for both DNNs before their error rates stabilize. The inverse design DNN, on the other hand, take much less training time due to the relative simple network architecture. Specifically, the training of the inverse design DNN converged after 10,000 iterations, which takes only 15 minutes. Learning curves recording the error history during the training of each network are shown in Fig. S1.

**Table S1. Hyperparameters used in the training of forward prediction and inverse design DNNs**

| Hyperparameters | Forward DNN  (real) | Forward DNN  (Imaginary) | Inverse design DNN |
| --- | --- | --- | --- |
| Training set size | *70,000* | *70,000* | *35,000* |
| Test set size | *30,000* | *30,000* | *15,000* |
| Optimizer | Adam | Adam | Adam |
| Learning rate | *10^-4^* | *10^-4^* | *10^-4^* |
| Batch size | *256* | *256* | *256* |
| Nonlinear activations | ReLU | ReLU | ReLU |
| Iterations | *10,000* | *10,000* | *10,000* |
| Time taken | *72 h* | *72 h* | *15 mins* |
| Error | *0.000308 (0.000735)* | *0.000326*  *(0.000788)* | *0.000068* |


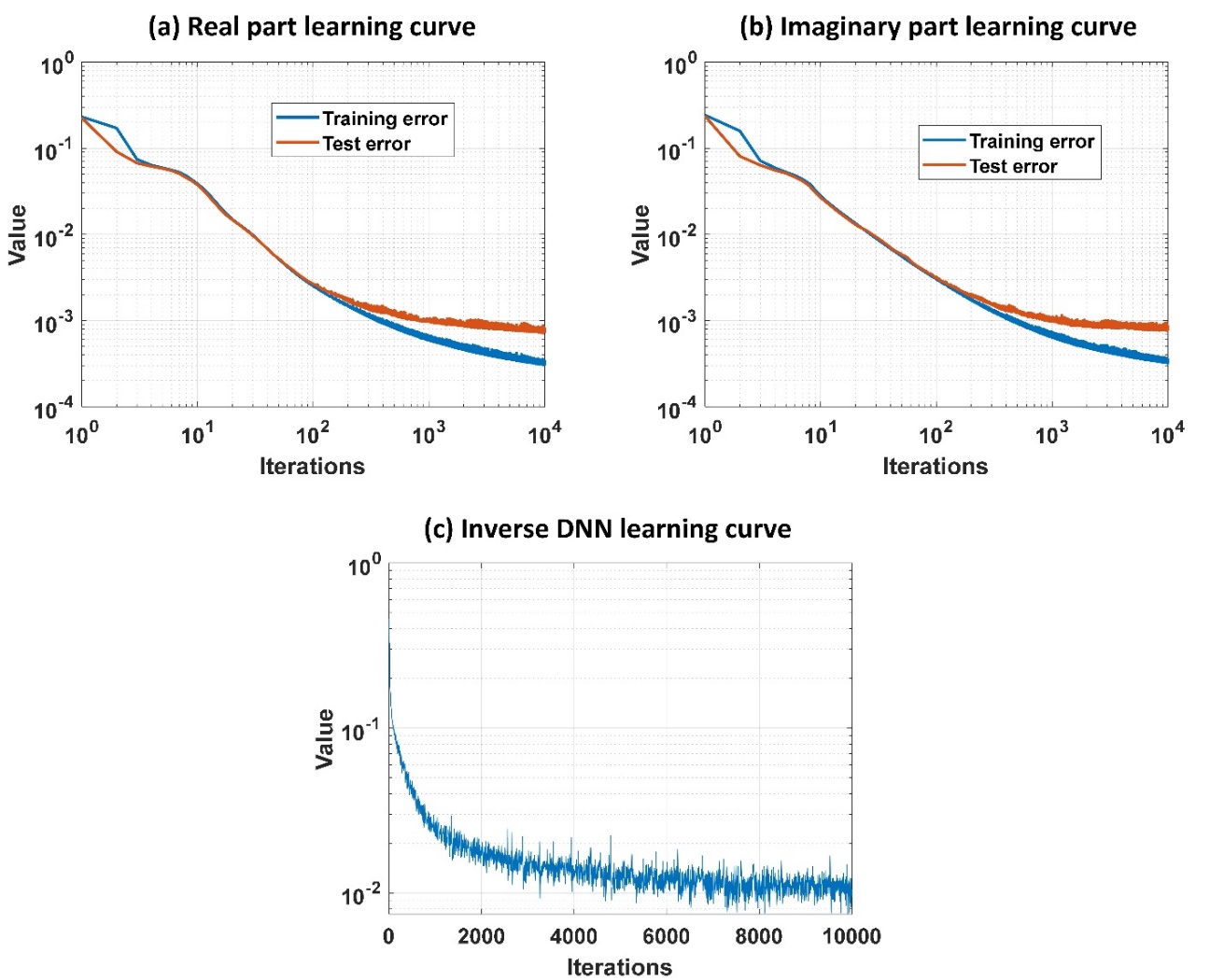


**Figure S1. Learning curves of the forward & inverse DNNs.** Showing in the figures are the learning curves of **a** real part DNN; **b** imaginary part DNN; **c** filter inverse design DNN.

**Section IV – Additional DNN prediction examples**

Here we have included additional examples to showcase the accuracy of the forward prediction DNN. In Fig. S2, four meta-atom designs with different shapes were randomly selected from the test dataset (shown on the left of each subplot). The real part (in blue) and imaginary parts (in red) of their complex S-matrices, including the S_11_, S_12_, S_21_, and S_22_ are shown on the right in each subplot. The dotted lines are the prediction results generated by the network, while the solid curves are the ground truth derived with the full-wave simulation tool.


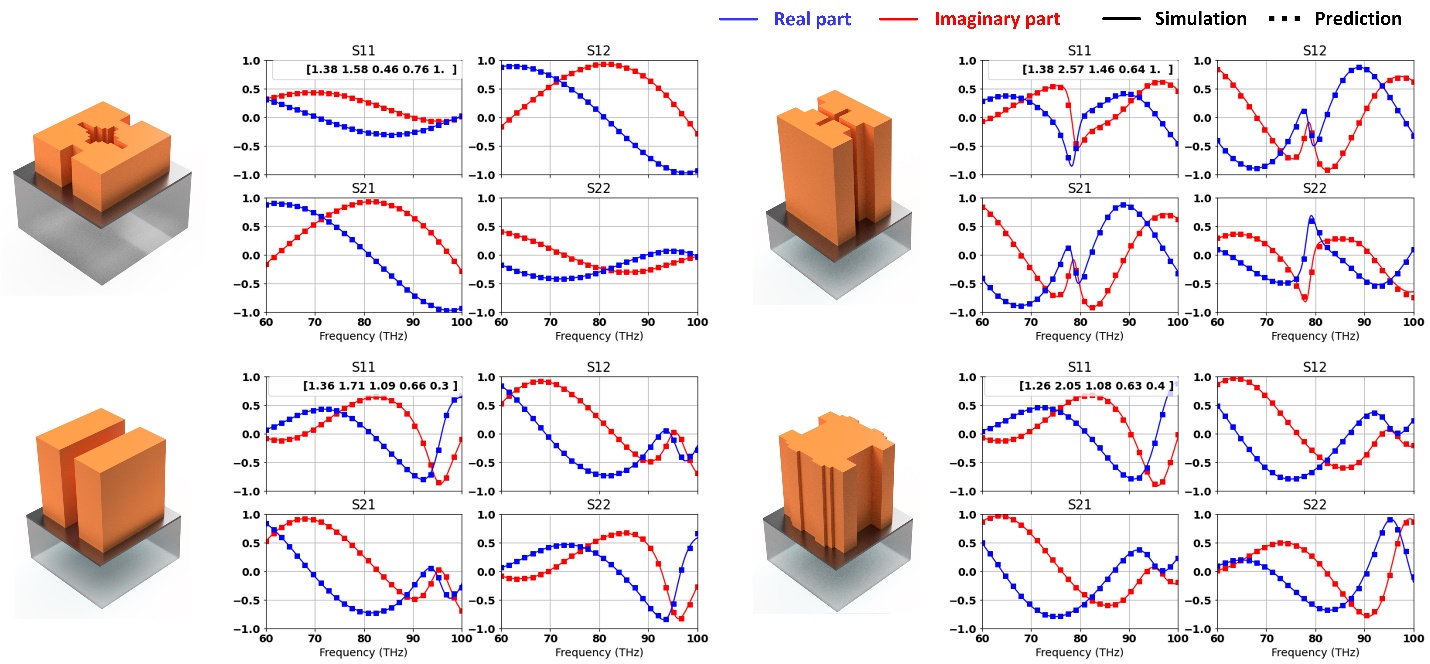


**Figure S2. Additional examples with the forward predicting DNNs.** Dotted lines represent the DNN prediction results, while solid curves are simulation results (ground truth). Parameters including lattice size, the thicknesses of MgF_2_, GSST meta-atom, and SiO_2_ substrate, as well as the crystallization states are shown on the top-right corner of the subplots (parameters listed in the same order as here; all lengths are in μm). 3-D models of each meta-atom are is shown on the left of each subplot.

Apart from the results shown in Fig. 3, we select another meta-atom design from the test dataset and employ the forward prediction network to evaluate its performance in different crystallization states (Fig. S3). The real part (in blue) and imaginary parts (in red) of their complex S-matrices, including the S_11_, S_12_, S_21_, and S_22_ are shown on the right in each subplot. The dotted lines are the prediction results generated by the network, while the solid curves are the ground truth derived with the full-wave simulation tool. Just to emphasize that ‘arbitrary’ optical responses are possible and not just bandpass filter transmission responses, we’ve also included the spectral transmission of the DBR-metasurface-DBR structure employing this particular meta-atom.


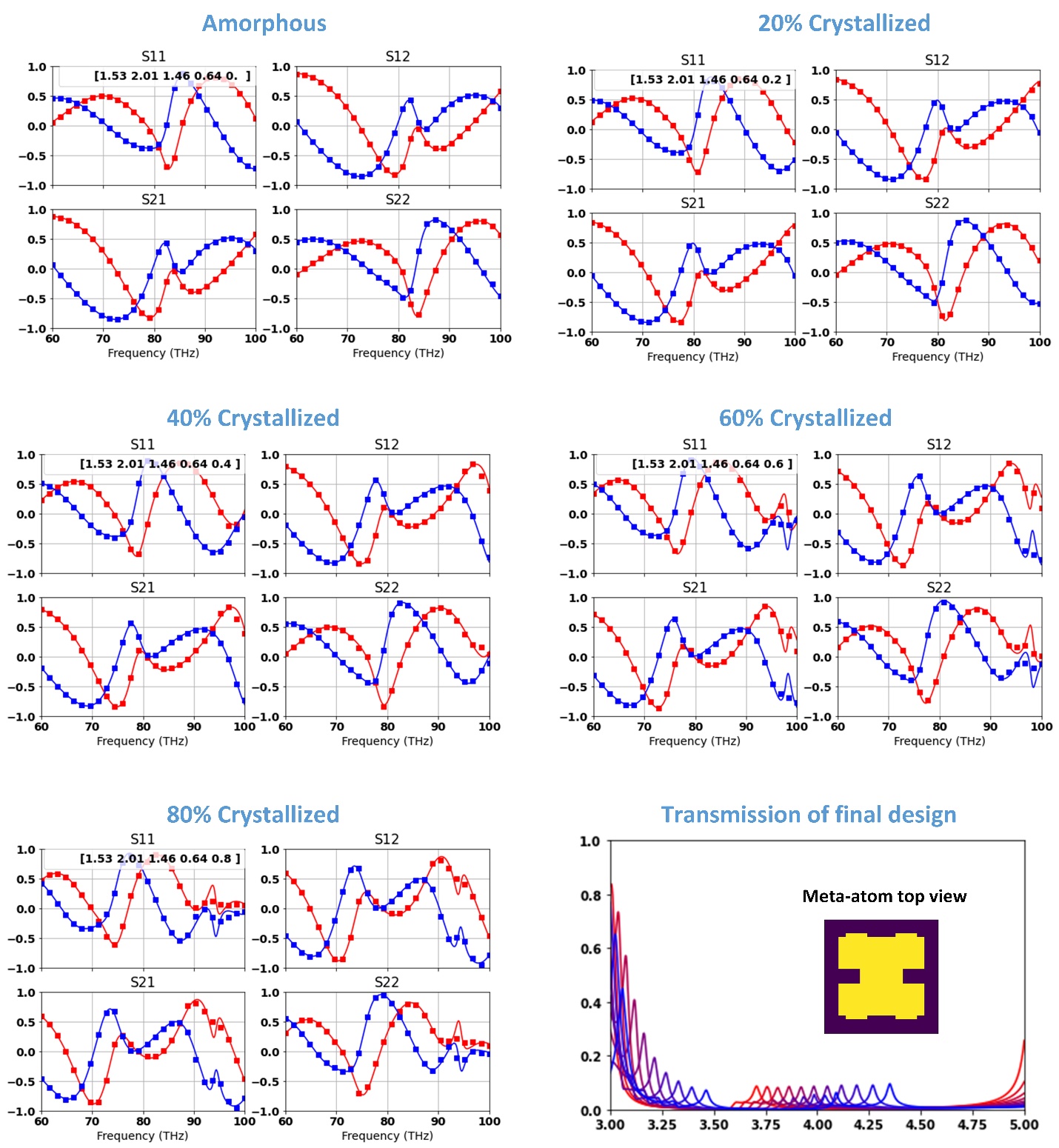


**Figure S3. An additional example to showcase the responses of meta-atoms in different crystallization states.** Dotted lines represent the DNN prediction results, while solid curves are simulation results (ground truth). Parameters including lattice size, the thicknesses of MgF_2_, GSST meta-atom, and SiO_2_ substrate, as well as the crystallization states are shown on the top-right corner of the subplots (parameters listed in the same order as here; all lengths are in μm).

**Section V – Inverse design performance characterization**

To better characterize the performance of the inverse designs showing in Fig. 5, here we have overlayed the S-matrices of the generated designs with the preset targets in each subplot to visualize the differences. As shown in Fig. S4, the schematics of the two designs are shown in Fig. S4a, while the real and imaginary part of the S-matrices of the two designs are showing in b and c, respectively. The target transmission (dotted lines in d) and the transmission spectra of the final designs (solid lines in d) are also included. The tuning range of the generated designs agreed well with the preset targets. Due to the reflection caused by the slight differences between the target S_11_/S_22_ and the result S_11_/S_22_, the transmission in the passbands are lower comparing to the design targets.


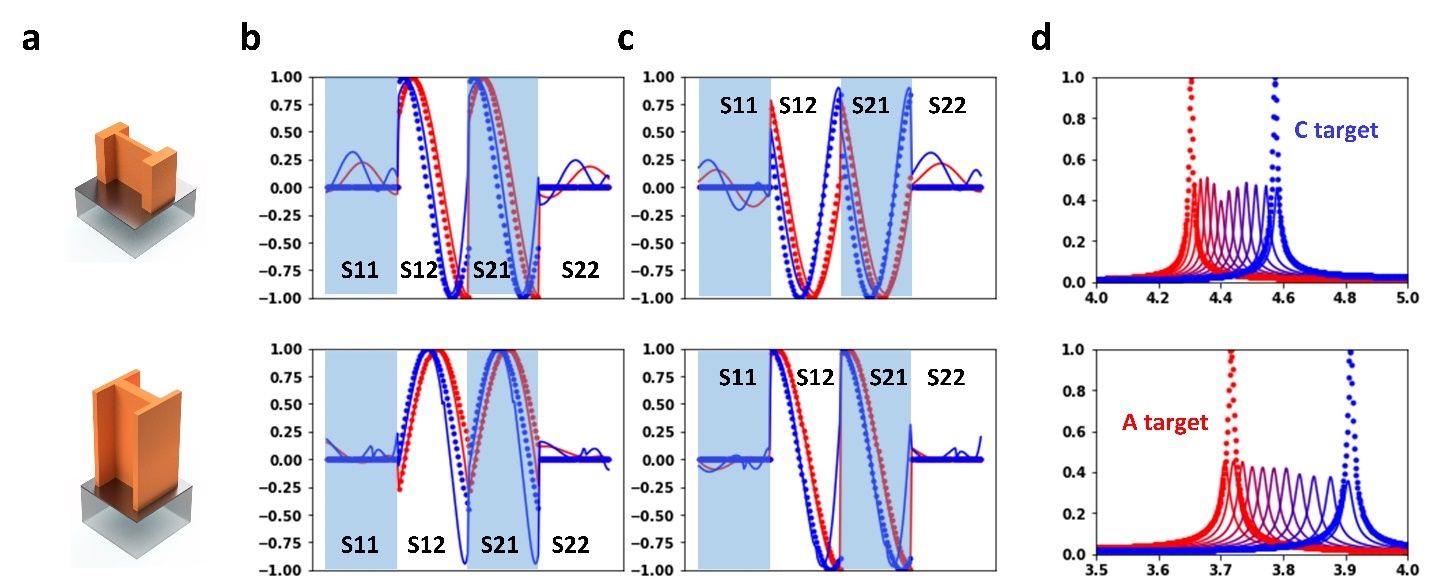


**Figure S4. Design examples with the inverse DNN (overlapped targets and results). a** Schematic of the generated designs. **b** Real part of the S-matrix (targets and results). **c** Imaginary part of the S-matrix (target and results). d Transmission spectra (target and results). In each subplot, solid curves represent the design targets, while the dotted lines represent the performance of generated designs.

Reference for SI

1. C. Ríos et al., "Ultra-compact nonvolatile photonics based on electrically reprogrammable transparent phase change materials," *arXiv preprint arXiv:.06010* (2021).
